# School personnel’s comprehension and perceptions of school recruitment materials for Strong Teens for Healthy Schools

**DOI:** 10.3389/fpubh.2026.1852763

**Published:** 2026-08-04

**Authors:** Alisha George, Andrew McNeely, Gabrielli T. De Mello, Cassandra M. Beattie, Chad D. Rethorst, Rebecca A. Seguin-Fowler, Jacob Szeszulski, Alexandra MacMillan Uribe

**Affiliations:** 1 Institute for Advancing Health Through Agriculture, Texas A&M AgriLife Research, Dallas, TX, United States; 2 School of Economic, Political, and Policy Sciences, University of Texas at Dallas, Richardson, TX, United States; 3 Institute for Advancing Health Through Agriculture, College Station, TX, United States

**Keywords:** administrators, adolescent health, health education, health promotion, school teachers

## Abstract

Recruiting for school health programs has become a challenge in recent years owing to multiple environmental, institutional, and logistical factors. The aims of this cross-sectional study, conducted at a professional health educators conference (K-12 schools), are to (1) assess comprehension of recruitment materials created for Strong Teens for Healthy Schools (STHS), a middle school-based program focused on physical activity and nutrition, and (2) understand perceptions of barriers to participating in STHS among school district administrators and staff. Participants (*n* = 28) were assigned to review recruitment materials and provide feedback on a flyer (*n* = 18) or two videos (*n* = 10) that explain STHS research and program activities. Interviews were deductively coded and thematically analyzed. Participants were primarily non-Hispanic (93%), White (71%), and female (83%), with 19.8 ± 9.8 years of experience in health education. Five themes emerged. (1) Participants recognized that the flyer emphasized the program’s focus on empowering students to make changes in nutrition and physical activity. (2) Participants desired more information on the flyer about program logistics to help them make an informed decision. (3) Videos were perceived to showcase research activities clearly. (4) Concerns were raised regarding the curriculum content and the role of health assessments in program success. (5) Buy-in from administrators, teachers, parents, and students could be potential barriers to implementing STHS. Findings from this study help inform the creation of recruitment materials for future health promotion programs.

## 1 Introduction

Schools serve as an essential platform for fostering positive youth development and outcomes (i.e., competence, confidence, connection, character, caring) (1–4) through consistent exposure to health promotion programs and social-behavioral learning (5–7). An evaluation of these health programs through research helps determine their effectiveness for student health and understand their adoption and implementation (8, 9). However, recruitment for school-based health research presents a challenge, due to the complex administrative processes researchers must navigate to work with schools as research sites and for program implementation (10, 11). Effective school recruitment relies on tailored informational materials that act as a strong first impression, generating interest and overcoming adoption barriers by aligning with the institution’s core values (12–14) Furthermore, new programs and/or research studies often include novel elements, which may create new barriers to adoption and further necessitate the development of tailored recruitment materials that are more attuned to their values and interests prior to use (12).

Strong Teens for Healthy Schools (STHS) is a newly developed middle school-based program that provides a critical opportunity to examine these recruitment dynamics in the context of a new school based heath intervention. Doubling as a research study, STHS aims to promote nutrition, physical activity, and positive youth development and was adapted from Change Club, a 24-week program designed to educate adults on healthy living and environmental civic engagement concepts (16–18). STHS’s 16-week curriculum engages students in planning and implementing physical activity and healthy eating-focused environmental change projects in their school environment (e.g., starting a new school garden, buying new physical activity equipment for their gym) (15–18). Participating schools receive $3,000 in project seed funding, and all lessons – available in both 30- and 60-min formats – are aligned with Texas Essential Knowledge and Skills [TEKS] standards for grades 5–8 (18). The program also offers flexible implementation options, including independent delivery by existing school staff and/or support from researchers and county extension agents (15). STHS aims to recruit schools with >40% of students who identify as Black or Hispanic to participate in a randomized controlled trial that measures indicators of metabolic syndrome (e.g., BMI, blood pressure, fasting glucose, cholesterol) (16, 17), dietary intake of fruits and vegetables (using the VeggieMeter) (18), physical activity (via a fitness tracker or PACER test), and positive youth development outcomes (1) to evaluate the effectiveness of the program.

Given the multicomponent nature of the STHS program, the materials used to engage potential school partners are critical to its adoption. Prior research has documented barriers to recruiting schools for health-based programs and research studies (11); however, there is a lack of research examining how school staff perceive and understand recruitment materials. Given the lack of research, there is a need for studies that assess the quality and content (e.g., images, text) of materials used to convey information about school-based research programs. The first aim of this study is to assess comprehension of recruitment materials created for the STHS program and research study. The second aim is to understand perceived barriers to enrolling and participating in STHS among school personnel, to inform the creation and enhancement of recruitment materials in future school-based health research initiatives.

## 2 Methods

### 2.1 Design

This qualitative cross-sectional study was conducted at an annual school health conference in Frisco, Texas, United States. Attendees (approximately 2,500) included potential adopters (e.g., school and district administrators) and implementers (e.g., school health staff) of STHS. Short intercept interviews were used to collect feedback from Texas educators about informational materials – flyers and videos – used to promote the STHS program and research study. The study was reviewed and determined to be exempt by the Institutional Review Board at Texas A&M University (STUDY2024-0512) since the research only included survey and interview procedures that were recorded by the investigator, such that the identity of the human subjects could not readily be ascertained, directly or through identifiers linked to the subjects and any disclosure of the human subjects’ responses outside the research would not reasonably place the subjects at risk of legal, financial, or reputational harm. Prior to data collection, the research team members were trained in how to conduct intercept interviews (15, 18). Researchers followed a structured interview guide (Supplementary Appendix 1) and a standardized program description to remove any biases in participant responses arising from the subjective delivery of the program.

### 2.2 Procedures

#### 2.2.1 Participant recruitment and enrollment

To recruit participants, three study team members were stationed in high traffic areas: two circulated the exhibit hall, while the third remained at a booth to answer questions and observe attendees. All three researchers used iPads to show participants the recruitment materials and QR codes which allowed participants to access survey questions when ready. Eligible participants were ≥18 years old and able to read and write in English.

To enroll in the study, participants provided verbal consent and were assigned a unique identification number. Participants then scanned a QR code to complete a brief demographic survey using REDCap, a data capture tool. Following this, participants reviewed the STHS recruitment materials by either reading a paper flyer or watching two videos consecutively on an iPad. Researchers alternated which material participants received; however, due to logistical constraints, only flyers were reviewed at the booth, resulting in more interviews focused on flyers than videos.

#### 2.2.2 Materials

The informational flyer and videos provided a comprehensive overview of STHS and its components, outlined eligibility and randomization, described health assessment activities, and highlighted the benefits to participating schools. The flyer was double sided and included information on the research activities in which parents and staff might be involved, along with financial compensation for various stakeholders participating in the study. The flyer also included a QR code that linked directly to the program application page with the research team’s contact information. While reviewing the flyer, participants could scan the QR code to view the application page, but they were not directed to do so; this allowed researchers to observe participants’ natural approach to reading the flyer. While the flyer provided a high-level summary, the accompanying videos offered a more detailed visual breakdown of the program. This information was divided into two parts: the first video introduced the research team and lead investigators to establish the study’s importance, while the second utilized staged footage of data collection procedures to enhance transparency regarding the health assessments. Both videos were about 2 min long and were hosted on YouTube.

#### 2.2.3 Data collection

After reviewing these materials, participants responded to two sets of interview questions. Responses were audio-recorded using a Sony ICD-UX570 digital voice recorder and participants were allowed to revisit the materials at any point during the interview. The first set of questions focused on comprehension of the material that was reviewed. After the comprehension questions, researchers provided a standardized description of the program, outlined the curriculum’s goals, key study points, and the financial compensation for schools that agreed to participate. Researchers then posed questions to assess program implementation within the participants’ school settings.

Interviews were designed to last approximately 10–15 min to accommodate the normal time between conference sessions. Participants were compensated with an STHS promotional item valued at up to $15 (e.g., mug, bag, water bottle). A process flow of data collection activities and the interview guide used can be found in Supplementary Appendix 2.

#### 2.2.4 Data analysis

Interviews were transcribed using NVivo software and individually reviewed for accuracy. Transcripts were uploaded into ATLAS.ti and coded by two researchers using an iterative categorization and deductive content analysis approach. Deductive codes were based on interview questions (described above) and were analyzed for patterns or similar ideas, which were then grouped into themes to represent the most prominent ideas in the data (22). Demographic characteristics were analyzed using descriptive statistics (distribution, measures of central tendency, and measures of variability) and were calculated in Excel.

## 3 Results

A total of 28 interviews were conducted, of which 18 participants viewed the flyer, and 10 viewed the videos. Of the video interviews, two were concluded early because participants had to attend their next conference session and did not return to finish them. Participants were primarily female, non-Hispanic White, with at least 3 years of experience in education (Table 1). Since the majority of participants were teachers, only one participant served as a school administrator, and no significant differences in responses were found across roles, results for all groups have been presented together. Five themes were derived from the data: two about the flyer, two about the videos, and one about perceived barriers to implementing STHS as a program and research study. No significant relationship was found between themes and therefore they are presented as stand alone.

**Table 1:** Participant characteristics (*N* = 28).

Variable	Values
Age (yrs ± SD)	48 ± 11.01
Gender, *n* (%)	
Female	24 (83)
Male	4 (14)
Race *n* (%)	
American Indian or Alaskan Native	1 (3.5)
Black or African American	7 (25)
White	20 (71.4)
Hispanic *n* (%)	
Yes	2 (7.1)
No	26 (92.9)
Years of experience *n* (%)	
0–5	3 (10.7)
6–10	2 (7.1)
11+	23 (82.1)
Role *n* (%)	
Teacher	25 (89)
Teaching Assistant	1 (4)
Other	2 (7.1)

*Other roles include department chair and Adapted Physical Education (APE) teacher.

### 3.1 Theme 1 (Flyer)

*Participants recognized that the flyer emphasized the program’s focus on empowering students to make changes in nutrition and physical activity.* Participants who reviewed the flyer valued the program’s emphasis on student empowerment to modify physical activity and healthy eating behaviors, as well as its applicability to real-world health issues (Table 2, Quote 1). Furthermore, they hoped that students would use knowledge gained through the program to take ownership and responsibility for improving their existing habits (Table 2, Quote 2). The availability of funding was seen as an essential asset for facilitating empowerment, especially in resource-constrained school environments. For example, a few participants mentioned budget constraints and the lack of infrastructure to support health programs in their school settings (Table 2, Quote 3).

**Table 2:** Representative quotes from flyer reviewers on their comprehension of STHS.

Quote #	Representative quote
Theme 1: Flyer emphasized the program’s focus on empowering students to make changes in nutrition and physical activity.	
1	A high point for me was a focus on nutrition and physical activity. I am a health educator for middle school… so that stood out.
2	Well, reading it (flyer), it looks like it’s about teen empowerment. Taking control of their health, what they eat, and their physical activity is empowering.
3	Also, to be compensated for groups where they do not have a lot of money really, you know, get $500 in a budget…
Theme 2: The flyer lacked sufficient information on research and curriculum logistics to help in decision-making.	
4	I was wondering is, who’s going to do the finger sticks? Would it be the physical education teacher? Because I know we are not certified in that. Or would it be the nurse on campus?
5	Maybe just how would these (health assessments) be administered? … Do the kids see it? Did the other children see it? Some of them may even have concerns with me (the teacher) seeing the results, you know. So, would it be a private thing?
6	Obviously, as an educator and a parent, I would want to know what type of information my student is giving for a study…is it going to be anonymous? Is their data, their information, going to be kept and collected and things like that?
7	I think just kind of how does it work? Is it digital? Is it something that they log their food? Do they log their activity?

### 3.2 Theme 2 (Flyer)

*Participants desired more information on the flyer about research and curriculum logistics to help them make an informed decision.* Participants felt there was insufficient information about the commitment the school would need to make to support the success of the research procedures evaluating the STHS program. Flyer reviewers felt that more information could be provided about what would be needed to make the health assessments successful (Table 2, Quote 4). Participants also raised concerns about the privacy of health information being collected, alluding to apprehensions that parents and students may have about these measures being conducted in large groups (Table 2, Quotes 5 and 6). Additionally, participants wanted to know more about the format of the curriculum, online or in-person, which was not clearly addressed in the flyer (Table 2, Quote 7).

### 3.3 Theme 3 (Video)

*Videos were perceived as clearly showcasing research activities and objectives.* Participants who viewed the videos were able to connect the importance of health assessments to the curriculum’s impact on overall health. Most participants emphasized the importance of the measurements being taken and accurately relayed the major points (Table 3, Quote 1) of the videos shown, including study objectives and timelines. A participant highlighted two primary outcomes of STHS — physical activity and healthy eating — emphasizing how it empowers students to make choices they can carry into adulthood (Table 3, Quote 2).

**Table 3:** Representative quotes from video reviewers on their comprehension of STHS.

Quote #	Representative quote
Theme 3: Videos were perceived as clearly showcasing research activities and objectives.	
1	It sounds like you look at nutrition, physical activity, and health to evaluate whether your program, what you are implementing, is successful or not. So you are tracking the kids over the course of, I guess, a year maybe…
2	Well, what I saw was Strong Teens sets up a time to come to the school to set up a scenario of different events like blood testing, checking up the physical fitness, and another one is nutrition assessments.
Theme 4: The video lacked clarity on the curricular approach to facilitate student learning, including how environmental change projects were assessed.	
3	…It did not really say how the kids were going to learn, like what type of curriculum, how they are going to learn what’s affecting them the right way or the wrong way.
4	I would be curious to know, like, who does that (assessment of project), who grades, what are the parameters for the project…And then how does that correlate to the health assessment?

### 3.4 Theme 4 (Video)

*There was a lack of clarity regarding the curricular approach to facilitate student learning, including how environmental change projects were assessed.* Participants felt that the videos did not clearly relay the format of the curriculum (e.g., online or in-person), with more than half of the participants wanting to know more about how content of the curriculum applies to students (Table 3, Quote 3), methods for program implementation, and how the health assessments would be assessed in the context of the environmental change project (Table 3, Quote 4). Participants also felt strongly that educators would appreciate more information on how the success of the environmental change project is measured.

### 3.5 Theme 5 (Research and program implementation)

*Buy-in from administrators, teachers, parents, and students could potentially be barriers to STHS research and program implementation.* Participants across both the flyer and video groups felt that the health assessments could be a barrier to the program’s acceptance by district administrators. More specifically, participants felt that administrators’ decisions would be affected by their perceived pushback from parents on the health assessments. (Table 4, Quote 1). For example, parental support for health assessments would be challenging due to concerns about data privacy, especially when conducted in a large-group setting (e.g., height and weight measurements). (Table 4, Quote 2).

**Table 4:** Representative quotes about perceived potential barriers to STHS.

Quote #	Representative quote
Theme 5: Buy-in from administrators, teachers, parents, and students could potentially be barriers to STHS research and program implementation.	
1	I do not know how our admin would feel about kids having outside testing. (…) you would have to get your administrators behind that research piece for sure. *- Video Reviewer*
2	So, we had some feedback that they did not want these things measured in front of the other kids for fear that they’d be like, What did you get? You know that kind of thing. *- Flyer Reviewer*
3	I think that sometimes the struggle with implementing new programs is timing, you know, where is it going to fit, how is it going to fit? And getting the district to agree to want to do something like this in the schools. *- Flyer Reviewer*
4	I think it may be just finding a facilitator to do it. Like, what would it run through? PE? Health? Extracurricular? *- Video Reviewer*
5	First and foremost, the barriers are going to be trying to corral kids and persuade them to come out and listen…because their kids do not want to do anything extra if they do not have to. I have a 12-year-old and a 15-year-old, so I know that if it’s not something they feel they are getting from, they may not do it. *- Flyer Reviewer*

Additionally, participants believed that administrators would find it cumbersome to review and approve new material, as it would add to their existing workload. They perceived that administrators would face challenges in finding the right time to implement it, either during or after school hours, and that the curriculum did not cohesively fit into existing health, science, and physical education curricula (Table 4, Quote 3). Participants also thought that teacher support and endorsement of the program could pose a challenge due to the lack of instructional support for after-school programming and the added responsibility of the curriculum. (Table 4, Quotes 4). Apart from parent and teacher buy-in, student engagement in the program could also be a barrier to its success, as students would not want to do anything that would add to their already busy schedules if they were not receiving something (monetary or intellectual) that they thought was valuable (Table 4, Quote 5).

## 4 Discussion

This qualitative study sought to understand school personnel’s comprehension of recruitment material developed for the STHS program and research study, and perceived barriers to enrolling and participating. Five themes emerged from the analysis: (1) Flyers were effective at outlining the programmatic elements of STHS, specifically student empowerment; however, (2) more information was needed regarding the research components of STHS for them to make an informed decision. (3) The videos, on the other hand, provided participants with a clear understanding of research goals and logistics; however, (4) they lacked information on the curriculum. Finally, (5) participants felt that stakeholder buy-in at multiple levels is required to successfully implement STHS.

Our findings indicate that no single recruitment tool was sufficient to convey a holistic picture of the STHS program, encompassing its multiple components. The flyer was successful in generating initial interest and emphasized the program’s focus on empowerment-based health behavior changes. Participants perceived that the curriculum addressed persistent gaps in existing health education that extend beyond the individual level to support sustained change in health behaviors. This aligns with prior research suggesting that environmental and policy interventions can have a larger impact on health behaviors (20). Additionally, ecological models of health promotion emphasize that structural factors like access to healthy food, physical activity equipment, and supportive institutional policies facilitate healthier behaviors, reinforcing the need to emphasize them in recruitment material (23). However, procedures involving sensitive health data (e.g., weight and waist circumference) were identified as a potential deterrent to participation among schools and students (24). highlighting the need to contextualize these activities with broader benefits of participation to help them understand the scope and potential impact of STHS.

Videos were praised for their clarity in explaining research objectives and assessment procedures. However, comprehension of curriculum components and the connection between outcome measures and program benefits was limited. This gap may reflect the structure of the recruitment videos, with one video focusing exclusively on research activities. Digital media and video-based recruitment strategies have been successfully used in health promotion programs to improve comprehension and engagement among potential participants (23). However, recruiting for school-based interventions differs from individual-level recruitment efforts, especially since it requires coordination and buy-in from multiple stakeholders. Therefore, recruiting materials created for such programs must address the informational needs and priorities of these different groups to facilitate enrollment, encourage adoption, and program retention.

One strategy to create tailored recruitment materials and connect with decision-makers is to collaborate with a school or community “champion” who helps research teams connect with schools (24). Collaborating with school champions or other personnel to co-develop or adapt recruitment materials may enhance their relevance and effectiveness, ultimately fostering broader stakeholder support. These findings suggest that recruitment materials may be more effective when used complementarily and paired with interpersonal engagement strategies that leverage existing relationships within the school community, rather than used independently.

This study has several strengths. Interviewers received training using a standardized protocol, minimizing biases that could arise from the subjective delivery of the program overview. Second, materials were reviewed by the intended audience, school personnel who could serve as program champions. Third, the demographic composition of our sample closely mirrors national teacher demographics of 80% of K–12 public school teachers identifying as non-Hispanic White and 77% are female (25) consistent with our sample population. This supports the transferability of our findings to the broader national teaching population, while highlighting the need for future work with more racially diverse representative samples similar to that of Texas. Limitations of this study include a sample that is predominantly comprised of teachers which limits the insight from school administrators who might have different feedback on the material, warranting further exploration in future studies. Finally, the intercept interview format constrained the depth of discussion and exploration of broader concepts.

The goal of this study is to inform future recruitment strategies for school-based health research initiatives. Practical ways to enhance recruitment that was inferred from our interviews include making the messaging more cohesive (Theme 2 and 4), embedding interactive elements that link both promotional materials (e.g., QR codes in flyers) (Theme 1 and 3), and sequencing recruitment with in-person meetings with the support of a school champion so that all aspects of the program and research study are addressed (Theme 5). These approaches may help ensure that recruitment materials clearly communicate both the educational and research components of the program while addressing the informational needs of multiple stakeholders. Ultimately, such strategies may support the development of cohesive, contextually relevant, and engaging recruitment approaches for schools considering participation in health promotion research.

## Data Availability

The raw data supporting the conclusions of this article will be made available by the authors, without undue reservation.
