# Supplementary material for: School personnel’s comprehension and perceptions of school recruitment materials for Strong Teens for Healthy Schools
Source: Front Public Health. 2026 Aug 4;14:1852763. doi: 10.3389/fpubh.2026.1852763 (PMC13481800; doi:10.3389/fpubh.2026.1852763)
Supplement: Supplementary file 1 [file Data_Sheet_1.docx]

“I am now going to show you some informational material about the Strong Teens for Healthy Schools program. Please take however much time you need to review this material, and then I will ask you some questions about it when you are ready.”

1. **BEGIN RECORDING, STATE ID NUMBER**: Thank you for [watching that video/looking at that flyer]. Based on what you saw, can you describe the Strong Teens for Healthy Schools program back to me?
2. What are some of your takeaways (high points/ interesting things to you) about the program?
3. What do you feel like you still need to understand about the program (what’s missing from these materials?)
4. Thanks for answering those questions. Now I will summarize the program and its high points for you to make sure you have all of the relevant info.
   a. **Strong Teens for Healthy Schools (STHS) is a program that teaches middle schoolers about healthy eating and the importance of exercise while fostering leadership and community engagement. The goal of this curriculum is to help improve the cardiovascular and metabolic health of students enrolled in the program. Some important aspects of this curriculum are:**
   b. Fully funded - $3,000 is given to schools to support healthy changes in the school environment. Additional curriculum resources are also provided
   c. Implementation Support - through XXXX extension agents
   d. Flexible Length- 16 curricular content hours that can be delivered as 30-minute or 1-hour sessions.
   e. Flexible timing of delivery- can be done as an afterschool program, elective class, a club, a supplement
   f. TEKS aligned- All the lessons are aligned with health and physical education TEKS for 5*th*, 6*th,* and 7*th* grade.
   g. Optional Paid Health Assessments- Students can learn about their health through assessments like blood pressure, height, weight, fruit and vegetable intake, glucose, cholesterol, waist circumference, and running tests. All assessments are optional, and results are shared with parents. Students will be compensated for participating in the health assessments.
5. Based on these materials, what would be some of the benefits of the program for your school?
   a. What would be some of the barriers your school would face when implementing the program?

| Declined | Accepted | |  | Email for interested |
| --- | --- | --- | --- | --- |
| Time | Time | ID |  |  |
|  |  | 1 |  |  |
|  |  | 2 |  |  |
|  |  | 3 |  |  |
|  |  | 4 |  |  |
|  |  | 5 |  |  |
|  |  | 6 |  |  |
|  |  | 7 |  |  |
|  |  | 8 |  |  |
|  |  | 9 |  |  |
|  |  | 10 |  |  |
|  |  |  |  |  |
|  |  |  |  |  |
|  |  |  |  |  |
|  |  |  |  |  |
|  |  |  |  |  |
|  |  |  |  |  |
|  |  |  |  |  |
|  |  |  |  |  |
|  |  |  |  |  |
|  |  |  |  |  |
