# Supplementary material for: School personnel’s comprehension and perceptions of school recruitment materials for Strong Teens for Healthy Schools
Source: Front Public Health. 2026 Aug 4;14:1852763. doi: 10.3389/fpubh.2026.1852763 (PMC13481800; doi:10.3389/fpubh.2026.1852763)
Supplement: Supplementary file 2 [file Data_Sheet_2.docx]

## Video

1. [Introduction to Strong Teens for Healthy Schools](https://youtu.be/2jIruoRVWMs)

2. [Research Activities in Strong Teens for Healthy Schools](https://youtu.be/KUElY_7Fr6s)
